# Do people with rheumatoid arthritis maintain their physical activity level at treatment onset over the first year of methotrexate therapy?

**DOI:** 10.1093/rheumatology/keab060

**Published:** 2021-02-19

**Authors:** James M Gwinnutt, Husain Alsafar, Kimme L Hyrich, Mark Lunt, Anne Barton, Suzanne M M Verstappen, the RAMS co-investigators

**Affiliations:** 1 Centre for Epidemiology Versus Arthritis, Centre for Musculoskeletal Research, Faculty of Biology, Medicine and Health, University of Manchester; 2 NIHR Manchester Biomedical Research Centre, Manchester University NHS Foundation Trust; 3 Centre for Genetics and Genomics Versus Arthritis, Centre for Musculoskeletal Research, Faculty of Biology, Medicine and Health, University of Manchester, Manchester Academic Health Science Centre, Manchester, UK

**Keywords:** rheumatoid arthritis, physical activity, exercise, epidemiology, socioeconomic status

## Abstract

**Objectives:**

To describe how many people with RA reduce their baseline physical activity level over the first year of MTX treatment, and which factors predict this.

**Methods:**

Data came from the Rheumatoid Arthritis Medication Study (RAMS), a prospective cohort of people with early RA starting MTX. Participants reported demographics and completed questionnaires at baseline, and 6 and 12 months, including reporting the number of days per week they performed ≥20 min of physical activity, coded as none, low (1–3 days) or high (4–7 days). The physical activity levels of participants over 12 months are described. Predictors of stopping physical activity were assessed using multivariable logistic regression.

**Results:**

In total, 1468 participants were included [median (interquartile range) age 60 (50, 69) years; 957 (65.2%) women]. At baseline, the physical activity levels of the people with RA were: none = 408 (27.8%), low = 518 (35.3%) and high = 542 (36.9%). Eighty percent of participants maintained some physical activity or began physical activity between assessments (baseline to 6 months = 79.3%, 6 months to 12 months = 80.7%). In total, 24.1% of participants reduced physical activity and 11.3% of participants stopped performing physical activity between baseline and 6 months (6 months to 12 months: 22.6% and 10.2%, respectively). Baseline smoking, higher disability and greater socioeconomic deprivation were associated with stopping physical activity.

**Conclusion:**

Many people with early RA were not performing physical activity when starting MTX, or stopped performing physical activity over the first year of treatment. These people may require interventions to stay active. These interventions need to be mindful of socioeconomic barriers to physical activity participation.

**Rheumatology key messages**

- Twenty-eight percent of people with RA performed no exercise when starting MTX.
- Ten percent of those exercising when starting MTX stopped over the first year.
- Socioeconomic deprivation predicted stopping exercise; interventions should be designed to mitigate socioeconomic barriers to participation.

## Introduction

Physical activity (including exercise [1]) provides benefits for people with RA in terms of stamina, muscle strength, pain and function [2–5]. This has led to EULAR recommending physical activity for all people with inflammatory arthritis [6]. While evidence from the Netherlands suggests that physical activity is increasing in RA [7], many people with RA do not meet physical activity guidelines [e.g. EULAR [6] or the World Health Organization (WHO) [8]]. A pan-European cross-sectional study of 5235 people with RA from 21 countries reported that only 13.8% of participants reported performing physical activity three or more times per week, and the majority of participants performed no regular physical activity each week (>80% in 7 countries, 60–80% in 12 countries, and 45% and 29% in the final two countries) [9]. A cross-sectional study from the UK reported that women with RA performed 40% less moderate-to-vigorous physical activity compared with healthy controls. Only half of the RA group met WHO guidelines, compared with 82% of controls [10]. A cross-sectional study of the general population of the UK (UK-Biobank) showed that low levels of physical activity were more prevalent in those with self-reported RA compared with controls [*N* (%): RA = 1010/4396 (23.0%), controls = 67 394/433 680 (15.5%)] [11]. Furthermore, another study reported that people with RA spent more time sedentary than matched healthy controls (71% *vs* 62% of the day) [12].

These cross-sectional studies show that many people with RA do not perform sufficient physical activity, but it is unclear whether these people have always performed less physical activity, or whether people reduce their physical activity in the first few years following symptom onset. A study of 617 Swedish people with RA showed that only 8% of participants reported being physically inactive 5 years prior to RA onset [13]. Therefore, it is likely that many people with RA are reducing their physical activity levels in response to the symptoms of RA. This has implications for interventions; it may be easier to intervene early and maintain existing physical activity levels rather than trying to promote physical activity once individuals have stopped. However, at present we do not know how many people with RA stop performing physical activity in the early stages following the onset of symptoms.

Furthermore, a greater understanding of the factors driving reductions in physical activity in RA is important for determining, first, the content of interventions aiming to maintain physical activity levels, and second, the group at greatest risk of stopping exercising in order to target such interventions towards them. Studies have demonstrated that several factors are associated with lower physical activity levels in people with RA. The UK-Biobank study reported that the number of comorbidities participants reported was associated with lower physical activity in those with RA, although reverse causality cannot be excluded [11]. A study of 41 people with RA from the USA reported that exercise time was related to exercise self-efficacy and inversely related to disease activity and disability [14]. The association between function, self-efficacy and exercise level has been shown in other US [15], South Korean [16], and Swedish [17] studies, as well as a systematic review [18]. However, it is unclear whether these factors are also associated with reductions in physical activity following symptom onset, as well as absolute levels of physical activity.

Therefore, the objectives of this study were (i) to describe how many people with early RA reduce their baseline physical activity level over the first year of treatment with MTX, and (ii) to assess factors associated with reducing physical activity level and stopping physical activity over the first year of treatment.

## Methods

Data for this analysis came from the Rheumatoid Arthritis Medication Study (RAMS) [19], a UK-based, multicentre prospective observational study of people with early RA recruited as they started MTX treatment for the first time. For the purpose of the current study, RAMS participants were included if they reported data on their physical activity level (see below) at baseline. Participants with established RA were excluded (established RA defined as having >24 months symptom duration at baseline). RAMS ethical approval was obtained from the National Research Ethics Service Central Manchester Research Ethics Committee (ref: 08/H1008/25) and all participants gave their written informed consent.

### Assessments

RAMS participants were assessed at baseline by research nurses working in participating rheumatology clinics (i.e. when they started MTX) and at 6 and 12 months follow-up, reporting demographics [age, gender, smoking status, ethnicity (coded as either white or non-white due to low numbers for each of the non-White ethnicities), height and weight], undergoing 28-joint swollen and tender joint assessments, and completing questionnaires. Each participant’s BMI was calculated from their height and weight and categorized using WHO cut-offs [20]. Each participant’s socioeconomic status was defined based on their postcode using the Index of Multiple Deprivation 2010 (IMD) [21], coded as quintiles of the total population with the lowest quintile as the most deprived. Participants also reported on comorbidities from a set list, which were categorized into no comorbidities, one comorbidity, or two or more comorbidities. Blood samples were taken at each assessment and stored in freezers at −80°C. RF status (Beckman Coulter BLOSR6x105 and ELISA Genie HUFI03136) was determined from baseline samples and CRP (Beckman Coulter BLOSR6X99 and ELISA Genie HUFI00088, UK; mg/l) measured from samples at each time-point.

Participants completed questionnaires at each assessment including the British version of the HAQ [22], pain, fatigue and patient global visual analogue scales (VAS) (range 0–100), the Hospital Anxiety and Depression Scale (HADS) (anxiety = HADS-A, depression = HADS-D) [23], and the Brief Illness Perceptions Questionnaire [24]. The 2-component [25] (swollen joint count and CRP) and 4-component [26] (swollen and tender joint counts, CRP, and patient global assessment VAS) DAS28 were calculated at each assessment.

### Physical activity

Participants completed three physical activity-related Likert Scale questions at each assessment: (i) ‘During the past month, on average, on how many days per week have you taken exercise that has lasted at least 20 minutes?’ (scale: none, 1 day, 2–3 days, 4–6 days, everyday); (ii) ‘During the past month, on average, on how many days per week have you taken exercise that has made you sweat?’ (same scale; used to capture data on high intensity physical activity); (iii) ‘In comparison to others of your own age, do you think your physical activity is:’ (scale: much less, less, the same, more, much more). The participants were stratified into three exercise groups at each assessment based on their answer to question one: no physical activity, low physical activity (1 day and 2–3 days) and high physical activity (4–6 days and everyday).

### Statistical analysis

Baseline demographics, physical activity and disease-related variables were summarized using descriptive statistics, for the whole cohort and stratified based on the three exercise groups. The levels of physical activity at 6 and 12 months are also reported using descriptive statistics, and the number of people who changed physical activity group between each assessment is described. To assess predictors of decreasing physical activity level, participants were categorized into those that decreased their physical activity level between two consecutive time-points (i.e. baseline and 6 months, or 6 months and 12 months) and those who maintained some physical activity or improved their physical activity level. Changes from high to low, high to no physical activity, or low to no physical activity categories were counted as decreases in physical activity. People who maintained some physical activity (low physical activity or high physical activity at two consecutive assessments) or those who improved their physical activity (changed from low to high, no physical activity to low or no physical activity to high physical activity categories) were combined and acted as the reference. People who consistently performed no physical activity were excluded from this analysis. A multivariable random effects logistic regression model was used to identify baseline predictors of decreasing physical activity. Candidate baseline predictors were: age, gender, symptom duration, ethnicity, IMD quintile, smoking status, BMI, DAS28, HAQ, pain VAS, fatigue VAS, HADS-A, HADS-D, RF status, number of comorbidities and illness perception. Participants were classified into two latent classes of illness perceptions using latent profile analysis, one class representing positive illness perceptions and the other negative [27]. To assess predictors of stopping exercise completely, the same analysis was performed, with the outcome being changing from either high or low physical activity category to no physical activity. The comparison group were those who maintained some physical activity (including those who changed from high to low physical activity) and those who improved. Multiple imputation was used to impute missing data for covariates included in regression analyses. Analyses were performed using R version 3.6.0 (packages: foreign, grid, gridExtra [28], htmlwidgets [29], networkD3 [30], reshape2 [31], tidyLPA [32], tidyverse [33], wesanderson [34]) and Stata version 14 (Stata Corp., College Station, TX, USA).

## Results

In total, 1468 people with RA were included in this analysis {median [interquartile range (IQR)] 50, 69; 65.2% of women}. The median symptom duration at baseline was 6.2 months (IQR 3.7, 10.7). The participants were overweight on average [median (IQR) BMI 27.2 kg/m^2^ (24.1, 31.2)] and had moderate disease activity at baseline [median (IQR) DAS28 4.2 (3.2, 5.2)]. Furthermore, the cohort had moderate levels of disability, pain and fatigue on average at baseline [median (IQR) HAQ 1.0 (0.4, 1.6); pain VAS 48 (25, 70); fatigue VAS 51 (23, 73)] (Table 1).

**Table 1:** Baseline characteristics, total cohort and stratified by baseline physical activity level

	Total cohort	No physical activity	Low physical activity	High physical activity	
	
Variable	Median (IQR)/ *N* (%) [% missing]	Median (IQR)/ *N* (%) [% missing]	Median (IQR)/ *N* (%) [% missing]	Median (IQR)/ *N* (%) [% missing]	*P*
*N*	1468	408 (27.8)	518 (35.3)	542 (36.9)	
Age, years	60 (50, 69) [0]	59 (50, 68) [0]	59 (48, 68) [0]	62 (51, 70) [0]	<0.001^a^
Female, *N* (%)	957 (65.2) [0]	285 (69.9) [0]	349 (67.4) [0]	323 (59.6) [0]	<0.001^b^
Symptom duration, months	6.2 (3.7, 10.7) [0]	6.1 (3.4, 10.0) [0]	6.4 (3.8, 11.5) [0]	6.2 (3.7, 10.2) [0]	0.114^a^
Ethnicity					
White	1380 (94.0)	373 (91.4)	486 (93.8)	521 (96.1)	
Non-white	69 (4.7)	31 (7.6)	25 (4.8)	13 (2.4)	
Missing	19 (1.3)	4 (1.0)	7 (1.4)	8 (1.5)	<0.001^b^
IMD quintile					
1 (most deprived)	141 (9.6)	53 (13.0)	45 (8.7)	43 (7.9)	<0.001^b^
2	252 (17.2)	90 (22.1)	70 (13.5)	92 (17.0)	
3	224 (15.3)	48 (11.8)	93 (18.0)	83 (15.3)	
4	320 (21.8)	85 (20.8)	116 (22.4)	119 (22.0)	
5 (least deprived)	289 (19.7)	68 (16.7)	106 (20.5)	115 (21.2)	
Missing	242 (16.5)	64 (15.7)	88 (17.0)	90 (16.6)	
Smoking status					
Never	572 (39.0)	147 (36.0)	213 (41.1)	212 (39.1)	<0.001^b^
Former	600 (40.9)	168 (41.2)	195 (37.6)	237 (43.7)	
Current	284 (19.3)	89 (21.8)	107 (20.7)	88 (16.2)	
Missing	12 (0.8)	4 (1.0)	3 (0.6)	5 (0.9)	
BMI, continuous	27.2 (24.1, 31.2) [8.3]	28.5 (24.8, 32.6) [9.1]	27.2 (24.3, 31.6) [8.1]	26.2 (23.5, 30.0) [7.9]	<0.001^a^
BMI categories					
Underweight	13 (0.9)	4 (1.0)	4 (0.8)	5 (0.9)	<0.001^b^
Normal	438 (29.8)	98 (24.0)	156 (30.1)	184 (33.9)	
Overweight	462 (31.5)	125 (30.6)	153 (29.5)	184 (33.9)	
Obese	433 (29.5)	144 (35.3)	163 (31.5)	126 (23.2)	
Missing	122 (8.3)	37 (9.1)	42 (8.1)	43 (7.9)	
DAS28	4.2 (3.2, 5.2) [5.9]	4.6 (3.7, 5.6) [5.9]	4.2 (3.2, 5.1) [6.6]	3.8 (3.0, 4.7) [5.4]	<0.001^a^
DAS28-2C	3.3 (2.2, 4.5) [5.1]	3.6 (2.5, 4.8) [4.7]	3.3 (2.2, 4.4) [5.6]	3.1 (2.0, 4.2) [5.0]	<0.001^a^
HAQ	1.0 (0.4, 1.6) [0.7]	1.5 (0.9, 2.0) [1.2]	1.0 (0.4, 1.5) [0.4]	0.8 (0.3, 1.3) [0.6]	<0.001^a^
Pain VAS (0–100)	48 (25, 70) [2.2]	59 (36, 77) [1.5]	47 (23, 69) [2.1]	43 (22, 65) [2.8]	<0.001^a^
Fatigue VAS (0–100)	51 (23, 73) [2.1]	61 (36, 76) [1.0]	50 (23, 72) [3.3]	44 (18, 70) [1.9]	<0.001^a^
HADS Depression	5 (2, 8) [1.3]	8 (4, 10) [2.7]	5 (2, 8) [0.8]	4 (2, 7) [0.7]	<0.001^a^
HADS Anxiety	6 (3, 9) [1.5]	7 (4, 11) [3.2]	6 (3, 9) [0.8]	5 (3, 9) [0.9]	<0.001^a^
RF					
Positive	744 (50.7)	219 (53.7)	253 (48.8)	272 (50.2)	0.005^b^
Negative	392 (26.7)	94 (23.0)	143 (27.6)	155 (28.6)	
Missing	332 (22.6)	95 (23.3)	122 (23.6)	115 (21.2)	
Comorbidities					
None	579 (39.4)	153 (37.5)	200 (38.6)	226 (41.7)	0.003^b^
1	477 (32.5)	126 (30.9)	170 (32.8)	181 (33.4)	
≥2	411 (28.0)	129 (31.6)	147 (28.4)	135 (24.9)	
Missing	1 (0.1)	0 (0)	1 (0.2)	0 (0)	

a Kruskal–Wallis test.

b Chi^2^ test.

DAS28-2C: 2-component DAS28; HADS: Hospital Anxiety and Depression Scale; IMD: Index of Multiple Deprivation; IQR: interquartile range; VAS: visual analogue scale.

**Fig. 1:**
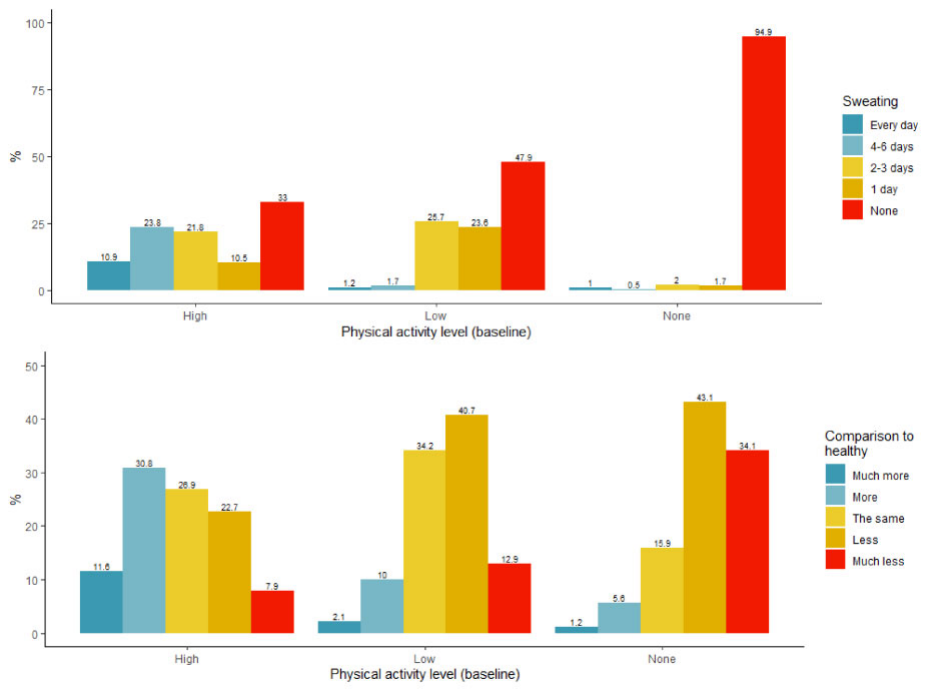
Self-reported physical activity level Number of days of physical activity causing sweat (**A**) and activity level in comparison to healthy people of similar age (**B**) at baseline, stratified by baseline activity group.

At baseline, 408 (27.8%) participants reported conducting no physical activity on average, 518 (35.3%) reported low physical activity levels (1–3 days per week) and 542 (36.9%) reported high physical activity levels (4–7 days per week). The level of physical activity was likely to be of predominantly moderate intensity, as just under half (47.9%) of those in the low physical activity group and 33% of those in the high physical activity group reported performing no exercise that caused sweating (Fig. 1A). The majority (69.3%) of those in the high physical activity group reported performing the same, more or much more physical activity compared with healthy people of a similar age, whereas the majority (53.6%) of people in the low physical activity group reported performing less or much less compared with healthy people of a similar age (Fig. 1B). A large proportion (77.2%) of those in the no physical activity group perceived performing less or much less physical activity compared with healthy people of a similar age.

The group who performed no physical activity at baseline had more women, more people reporting being of non-White ethnicity, higher BMI, lower socioeconomic status, more severe disease activity, more comorbidities and higher scores on the patient-reported outcomes compared with the other physical activity groups (Table 1).

### Changes in physical activity level over the first year of treatment with MTX

**Fig. 2:**
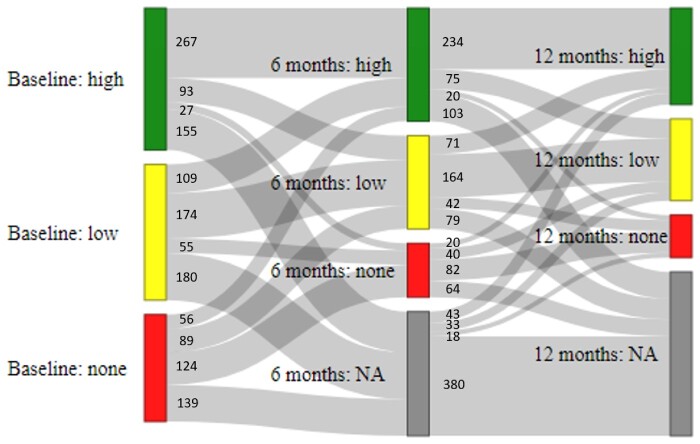
Changes in physical activity level over the first year of treatment with MTX NA: missing data.

The majority of participants who were seen at 6 months stayed in the same physical activity category as baseline [565/994 (56.8%)]. Four-fifths of the participants [788/994 (79.3%)] either maintained some physical activity (maintained high, maintained low or moved from high to low; *N* = 534) or improved their physical activity level (moved from none to low, none to high, or low to high; *N* = 254) over the first 6 months of treatment. The most common change from baseline to 6 months was a change from low physical activity to high physical activity [109/994 (10.9%)]. Of those performing physical activity at baseline, 24.1% (175/725) reduced their physical activity by 6 months, with 11.3% (82/725) stopping physical activity completely (Fig. 2).

Again, the majority of participants seen at 12 months stayed in the same physical activity category as at 6 months [480/748 (64.2%)]. Four-fifths of participants [604/748 (80.7%)] either maintained some physical activity (maintained high, maintained low or moved from high to low; *N* = 473) or improved their physical activity level (moved from none to low, none to high, or low to high; *N* = 131) between 6 and 12 months. The most common change between 6 and 12 months was from the high to low category [75/748 (10.0%)], followed closely by the number of people switching from low to high [71/748 (9.5%)]. Of those performing physical activity at 6 months, 22.6% (137/606) reduced their physical activity by 12 months, and 10.2% (62/606) stopped physical activity completely (Fig. 2).

### Predictors of decreasing physical activity level

Baseline predictors of reducing physical activity level compared with people who maintained or improved their activity level over the first year of MTX treatment were age [odds ratio (OR) 0.99 per year increase (95% CI 0.98, 1.00)], disability [HAQ; OR 1.36 per unit increase (95% CI 1.02, 1.80)] and being a current smoker [OR 1.47 current *vs* never smokers (95% CI 0.99, 2.18)] (Table 2). Lower levels of deprivation were numerically associated with lower odds of reducing physical activity over 1 year, but the associations were not statistically significant (Table 2).

**Table 2:** Baseline predictors of reducing physical activity level

Predictor	Reducing physical activity level^a^ [OR (95% CI)]	Stopping physical activity [OR (95% CI)]
Age	0.99 (0.98, 1.00)	0.99 (0.97, 1.02)
Men *vs* women	0.89 (0.66, 1.19)	0.48 (0.22, 1.01)
Symptom duration	1.00 (0.98, 1.03)	0.93 (0.87, 0.99)
Smoking status		
Ex *vs* never	1.09 (0.81, 1.48)	1.24 (0.60, 2.54)
Current *vs* never	1.47 (0.99, 2.18)	5.83 (1.98, 17.20)
Non-white *vs* white	0.62 (0.26, 1.47)	0.34 (0.04, 2.89)
IMD quintile		
1 (most deprived)	Ref.	Ref.
2	0.87 (0.51, 1.49)	0.58 (0.17, 1.95)
3	0.66 (0.38, 1.15)	0.26 (0.07, 0.97)
4	0.66 (0.39, 1.10)	0.21 (0.06, 0.75)
5 (least deprived)	0.80 (0.48, 1.35)	0.32 (0.09, 1.11)
BMI	1.00 (0.98, 1.03)	1.02 (0.97, 1.08)
HAQ	1.36 (1.02, 1.80)	2.43 (1.20, 4.91)
DAS28	0.98 (0.87, 1.11)	1.05 (0.79, 1.39)
Pain VAS		
Natural scale	1.00 (0.99, 1.01)	1.01 (0.99, 1.02)
Standardized scale	0.96 (0.79, 1.16)	1.16 (0.73, 1.82)
Fatigue VAS		
Natural scale	1.00 (1.00, 1.01)	1.00 (0.98, 1.01)
Standardized scale	1.07 (0.89, 1.29)	0.97 (0.62, 1.52)
HADS-A	1.00 (0.95, 1.04)	0.92 (0.82, 1.02)
HADS-D	0.99 (0.94, 1.04)	1.01 (0.89, 1.14)
Negative *vs* positive illness perceptions	1.00 (0.72, 1.38)	0.99 (0.45, 2.15)
Comorbidities		
1 *vs* none	0.91 (0.67, 1.25)	0.76 (0.36, 1.61)
≥2 *vs* none	1.07 (0.75, 1.52)	0.94 (0.41, 2.13)
RF+ *vs* RF-	1.00 (0.74, 1.36)	0.73 (0.35, 1.53)

a Reducing physical activity includes both reductions from high to low physical activity and stopping physical activity completely.

HADS: Hospital Anxiety (HADS-A) and Depression (HADS-D) Scale; IMD: Index of Multiple Deprivation; OR: odds ratio; VAS: visual analogue scale.

Baseline predictors of stopping physical activity completely were similar, but the effect sizes were stronger. Current smokers had >5-fold increased odds of stopping physical activity over the first year of MTX therapy compared with never-smokers [OR 5.83 (95% CI 1.98, 17.20)] and each unit increase in HAQ was associated with a >2-fold increase in odds of stopping physical activity [OR 2.43 (95% CI 1.20, 4.91)]. Socioeconomic deprivation was also strongly associated with stopping physical activity altogether over follow-up (Table 2). Lastly, men were less likely to stop physical activity compared with women.

## Discussion

This large cohort study of people with early RA has shown that the majority of participants reported performing some physical activity when starting MTX, although 28% of participants reported no physical activity. During the first year of treatment with MTX, 80% of participants were able to start or maintain some physical activity, even if some reduced their activity from high to low levels. This physical activity was likely to be low-to-moderate intensity, given the reports of low average number of days per week participants performed exercise that caused them to sweat. However, between a fifth and a quarter of participants who performed physical activity reduced their physical activity between each assessment, with around 10% of participants stopping physical activity altogether. Key socioeconomic indictors (smoking, socioeconomic deprivation) predicted stopping physical activity, as well as increased disability.

A similar distribution of physical activity levels to the current study was reported in a large cross-sectional study in the UK [11], with around a third of participants in each category. The high proportion of people with RA who do no physical activity when starting MTX treatment is concerning, given the known benefits of exercise with regards to general health and disease-related outcomes [2–5]. Potentially, interventions aiming to encourage people with RA to start exercising need to be delivered to these people at or close to the start of treatment [35], as it may become progressively harder to start physical activity as disease progresses.

Despite 28% of participants reporting no exercise at baseline, there was a high proportion of participants maintaining at least low physical activity levels over time. This has been reported in other studies, such as a study of 2752 Swedish people with prevalent RA which reported that the majority of participants (80%) had stable levels of physical activity over 2 years of follow-up [17]. Furthermore, 20–25% of participants increased their physical activity levels between assessments, potentially in reaction to improving symptoms due to successful treatment.

However, there was a significant proportion of participants who reduced their physical activity or stopped physical activity altogether over follow-up. Perhaps unsurprisingly, those with higher disability were more likely to reduce and stop physical activity over follow-up, an observation demonstrated in previous studies. For instance, participants in one study with a high baseline HAQ score (score from 1.1 to 3 out of 3) had 72% lower odds of being in the high physical activity group compared with the low physical activity group over 2 years of follow-up [OR 0.58 (95% CI 0.34, 0.96)] [17]. On the other hand, our study found no association between baseline multimorbidity and odds of reducing physical activity, despite studies reporting a correlation between number of comorbidities and physical activity level [11]. Potentially people with multimorbidity at baseline in this study had already reduced their physical activity level in response to the development of other health conditions (as seen by the higher number of comorbidities in the no physical activity group), and therefore did not reduce their physical activity level in the early phases of their RA.

Our study also illustrated the large role socioeconomic deprivation likely has on physical activity participation, with both smoking and IMD quintile strongly predicting reducing and stopping physical activity over follow-up. People in the general population with lower socioeconomic status are more likely to perform less physical activity [36], people with RA who had lower education were less likely to use physiotherapy services [37] and those people with RA who were employed were more likely to meet physical activity recommendations [15]. This contrasts with a 2014 systematic review which reported that many studies found no correlation between education, job status and physical activity [38].

People with RA may struggle to start or continue performing the recommended level of physical activity due to the significant barriers they face [5], and these barriers are likely to be higher for those with lower socioeconomic status. Some of these are similar to barriers faced by members of the general public, such as lack of time, motivation and the cost of exercise [16, 39]. People with RA also suffer disease specific barriers, such as lower functional ability, as highlighted in the current study, as well as a lack of knowledge and advice on whether exercise is safe and what exercise to perform [39]. Furthermore, these barriers are likely to increase as people move from early to established RA [40, 41]. Therefore, from a public health perspective, interventions aiming to help maintain or begin physical activity in RA should be delivered early in the disease course and may best be targeted towards those with lower socioeconomic status, given these people are most likely to stop physical activity. Furthermore, these interventions should be designed to mitigate socioeconomic barriers to participation, such as high cost, lack of childcare, lack of time and lack of awareness [42]. In addition, qualitative studies of people with RA show that physical activity maintenance strategies should focus on providing support and monitoring to help people make positive changes in their lives with appropriate incentives, developing communities for mutual support, and increasing people with RA’s feelings of autonomy and independence [43, 44].

Our study has a number of strengths. It is a large cohort study of people with early RA who are all at the same point in their disease history, namely starting MTX treatment for the first time. Therefore, the population that the findings from this study are applicable to is readily identifiable.

Limitations include the fact that physical activity was self-reported, meaning that there may be variation in the way people reported their physical activity level. The strong correlation between the three physical activity variables suggests that people’s ranking of physical activity level was relatively reliable, even if the absolute level of physical activity may be inaccurate. However, some people may have reported pre-RA exercise rather than current exercise at treatment onset. The correlation between disease activity and symptoms at baseline suggests this may not be the case. The participants were asked to recall their physical activity level over the previous month, a relatively long interval particularly during the early phases of RA. This was chosen to identify participants’ recent physical activity levels, but to avoid the influence of weeks in which, by chance, the participants experienced abnormally high or low physical activity just before assessments. The lack of a non-RA comparison group means it is difficult to assess whether the people with RA in this cohort were performing less physical activity than otherwise healthy people of a similar age, although previous research has shown this to be the case in general [10, 11]. Furthermore, there was no measure of self-efficacy, which has been shown to be associated with physical activity level in the past [14, 18, 45], and therefore self-efficacy could not be included in the analyses. Lastly, the physical activity categories (none, low, high) were quite wide, and therefore smaller changes in physical activity levels would not be included in the analyses. The decision to group the participants into three physical activity categories was made for the sake of power, to avoid having many small groups of physical activity change.

In conclusion, this study demonstrates that the majority of people with RA are performing some physical activity as they start MTX therapy, and that many people are able to start or maintain some physical activity over the first year of treatment. However, a significant proportion of people with RA performed no physical activity, and some people stopped performing physical activity completely over follow-up. These groups may need interventions to keep them physically active. Higher disability and increased socioeconomic deprivation were associated with reducing and stopping physical activity. This illustrates the societal barriers impeding people with RA from continuing to perform physical activity after starting treatment, and public health strategies aiming to maintain or promote physical activity in RA need to take socioeconomic barriers into consideration when designing and delivering interventions.
